# Risk-estimation and handling of occupational blood exposure accidents by nationally operating telephone service in the Netherlands

**DOI:** 10.1186/1753-6561-5-S6-P216

**Published:** 2011-06-29

**Authors:** PM Schneeberger, A Meiberg, J Warmelts, AC Leenders, P Van Wijk

**Affiliations:** 1 Medical Microbiology and Infection Control, Jeroen Bosch Hospital, 's-Hertogenbosch, Netherlands; 2 ArboNed/Keurcompany, Utrecht, Netherlands; 3 Medical Microbiology, Academic Medical Centre Amsterdam, Amsterdam, Netherlands

## Introduction / objectives

Health care workers, who are not working in a hospital, may not easily find access to care after having been exposed to blood exposure accidents. To improve handling of these accidents outside the hospital setting, in 2006 a nationally operating telephone expert centre was established by a Dutch occupational service.

## Methods

All occupational accidents could be reported directly by the injured to a national call centre on 24/7 basis. Trained staff members provided an assigned risk-estimation and plan of action to take appropriate measures, whenever indicated. All registered accidents in a 3-year period were analyzed on incidence and risk level per occupational branch. Also hepatitis B vaccination level, interval between accident and reporting, and the medical interventions taken were analyzed.

## Results

A total of 2927 reported accidents were analysed. Private clinics and hospitals had highest incidence rates (68.5 and 54.3 accidents/1000 person years, respectively). Ambulance staff, midwife practices and private clinics reported more high risk accidents whereas penitentiary institutions often reported accidents with no risk at all. Hepatitis B vaccination levels in mental health care, penitentiary institutions and cleaners was low (<70%). Mental healthcare staff as well as private clinics and midwifes often reported late.

## Conclusion

The nationally operating centre proved to be capable of organizing the handling of blood exposure accidents. Several occupational branches, in which the risks so far were not fully known, showed to have a serious risk for blood exposure accidents. In these branches more research is needed to explore these risks and design preventive measures.

## Disclosure of interest

None declared.

